# Polyurethane sheet: A potential substitute of surgical cotton gauze

**DOI:** 10.1186/1749-8090-6-26

**Published:** 2011-03-07

**Authors:** Takeshi Shimamoto

**Affiliations:** 1 Department of Cardiovascular Surgery, Kurashiki Central Hospital,Kurashiki, Japan

## Abstract

Polyurethane sheet is a pure white, soft, and flexible synthetic sponge with a mechanical strength and abrasion resistance equivalent or even superior to other sponge materials. Our study indicates that the blood absorption power of the polyurethane sheet is equivalent to that of the cotton gauze even after repeated use, and it has the potential to decrease the total amount of sponge usage. Further, accurately identifying the bleeding point might be easier with the polyurethane sheet.

## Findings

Our study indicates that the blood absorption power of the polyurethane sheet is equivalent to that of the cotton gauze even after repeated use, and it has the potential to decrease the total amount of sponge usage. Further, accurately identifying the bleeding point might be easier with the polyurethane sheet.

## Text

The uses of surgical gauze can be broadly classified as absorption of blood and identification of the bleeding site. In cardiothoracic surgery, significant amounts of gauze are used in each case because once a gauze piece gets soaked in blood, it never regains its original whiteness and its absorption power despite a thorough rinse with water and a subsequent squeeze. Its consumption may increase enormously, particularly during aortic surgeries that involve intractable bleedings. In such cases, counting the used gauze pieces before wound closure might be troublesome for the nursing staff or surgical technicians; as a result, the count can be inaccurate and cause potential postoperative accidental sponge retention [1].

Polyurethane sheet is a pure white, soft, and flexible synthetic sponge with a mechanical strength and abrasion resistance equivalent or even superior to other sponge materials. It is stable against acidic or alkaline detergents. Since no toxic agents such as formalin are used during its manufacture, its intraoperative use is considered safe.

**Figure 1:**
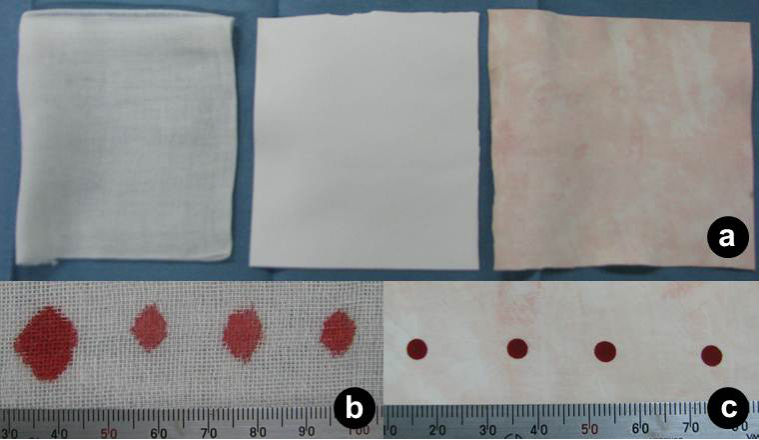
**(a) An unused surgical cotton gauze (left), unused polyurethane sheet (center), and polyurethane sheet subjected to the blood-soak and water-rinse procedure 10 times (right)**. Note that the polyurethane sheet retains sufficient whiteness even after repeated use. Four drops (0.1 cc each) of blood were dropped at 2-cm intervals on a new surgical cotton gauze (b) and a polyurethane sheet that was used 10 times (c). Note that the spread of the blood is lesser on the polyurethane sheet.

The major advantage of polyurethane sheet is its ability to retain fluid even after repeated use. To investigate the feasibility of its use as surgical gauze, 6 pairs of conventional cotton surgical gauze (EB sterile; Kawamoto Corporation, Osaka) and 2-mm-thick polyurethane sheet (Sofrous SK; Aion Corporation, Osaka) were compared. Both these sponges, which had identical weight, were prepared as 15 × 15-cm pieces, and then soaked in canine heparinized blood. The absorption ratio (weight of the maximum blood absorbed by the sponge/dry weight of the sponge.) was 6.13 ± 0.16 for the cotton sponge and 6.49 ± 0.18 for the polyurethane sheet; the difference was not statistically significant (Mann-Whitney U test; SPSS^® ^version 10; Illinois, Chicago). The polyurethane sheets were then rinsed with 500 cc of water, squeezed manually, and resoaked in the canine heparinized blood. This procedure was repeated 10 times without changing the water used for rinsing. At this stage, the absorption ratio for the polyurethane sheet was 6.55 ± 0.28 (the difference was not significant when compared with the absorption ratios of the sheet when first used and of the cotton gauze), indicating that the blood absorption power of the sheet remained unchanged even after repeated use and was equivalent to that of the cotton gauze. Moreover, the whiteness of the sheet was almost completely maintained after rinsing and squeezing (Figure 1a). Subsequently, 4 drops (0.1 cc each) of blood were dropped on both the materials at 2-cm intervals, and the manner of blood spread was observed: the spread was lesser and the demarcation was more evident on the polyurethane sheet (Figure 1b,1c).

This study indicates that the blood absorption power of the polyurethane sheet is equivalent to that of the cotton gauze even after repeated use, and it has the potential to decrease the total amount of sponge usage. Further, accurately identifying the bleeding point might be easier with the polyurethane sheet. Although a future study regarding the safety and feasibility of reuse is warranted, polyurethane sheets may function as a substitute of conventional surgical gauze, thus facilitating a more efficient surgery.

## Competing interests

None: Dr.Shimamoto has no commercial or financial relationship with any company manufacturing polyurethane sheet, and swear that this study was conducted from his own scientific interest.
